# Mathematical and Computational Modelling of Ribosomal Movement and Protein Synthesis: an overview

**DOI:** 10.5936/csbj.201204002

**Published:** 2012-02-20

**Authors:** Tobias von der Haar

**Affiliations:** a School of Biosciences and Kent Fungal Group, University of Kent, Canterbury, CT2 7NJ, UK

**Keywords:** Translation, Polysome, Biosynthesis, mRNA, Ribosome, Gene expression

## Abstract

Translation or protein synthesis consists of a complex system of chemical reactions, which ultimately result in decoding of the mRNA and the production of a protein. The complexity of this reaction system makes it difficult to quantitatively connect its input parameters (such as translation factor or ribosome concentrations, codon composition of the mRNA, or energy availability) to output parameters (such as protein synthesis rates or ribosome densities on mRNAs). Mathematical and computational models of translation have now been used for nearly five decades to investigate translation, and to shed light on the relationship between the different reactions in the system. This review gives an overview over the principal approaches used in the modelling efforts, and summarises some of the major findings that were made.

## Introduction

**Figure 1:**
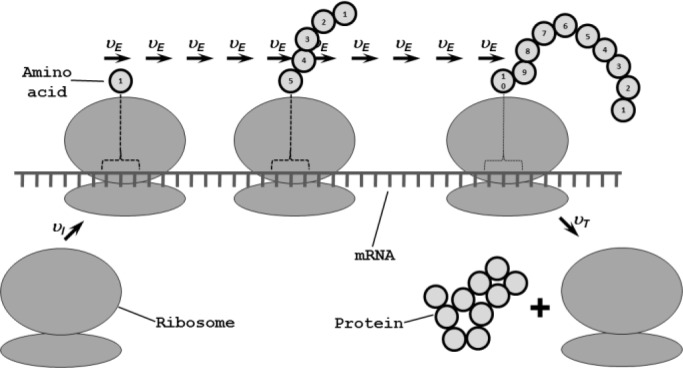
**A coarse-grained model of translation**. The depicted model is similar to the model used in the earliest modelling studies (eg ref. 49). Ribosomes assemble onto the mRNA with a rate *υ*_*I*_; proceed along the mRNA in one codon-steps with a uniform rate *υ*_*E*_, incorporating an amino acid at every step; and finally leave the mRNA from the last codon with a rate *υ*_*T*_. The model can be made more realistic by assuming different *υ*_*E*_ for the different codons.

Translation or protein synthesis involves the decoding of a linear template (the mRNA) by molecular machines (the ribosomes). Translation is conceptually divided into three sub-processes (Figure 1). During *translation initiation*, contacts are established between a ribosome and an mRNA. During *translation elongation*, the ribosome moves along the mRNA in one-codon steps, adding one amino acid to the growing polypeptide with each step. During *translation termination*, the complex of mRNA, ribosome and newly formed protein is dissolved. The molecular details of these three processes have been reviewed in depth [1].

Because of the directional movement of the ribosome, one mRNA can be translated by multiple ribosomes, thus forming a structure that is commonly termed a polyribosome or polysome (Figure 1). The idea that protein synthesis involves directional ribosomal movement along an mRNA molecule, then known as the Warner-Rich model, had been proposed in 1962 [2], and details of this model emerged through work from several groups [3, 4]. It was quickly realised that, although the rules that govern protein synthesis according to the Warner-Rich model are simple, these simple rules engender complex systems behaviour which cannot be intuitively understood [eg ref. 5].

**Figure 2:**
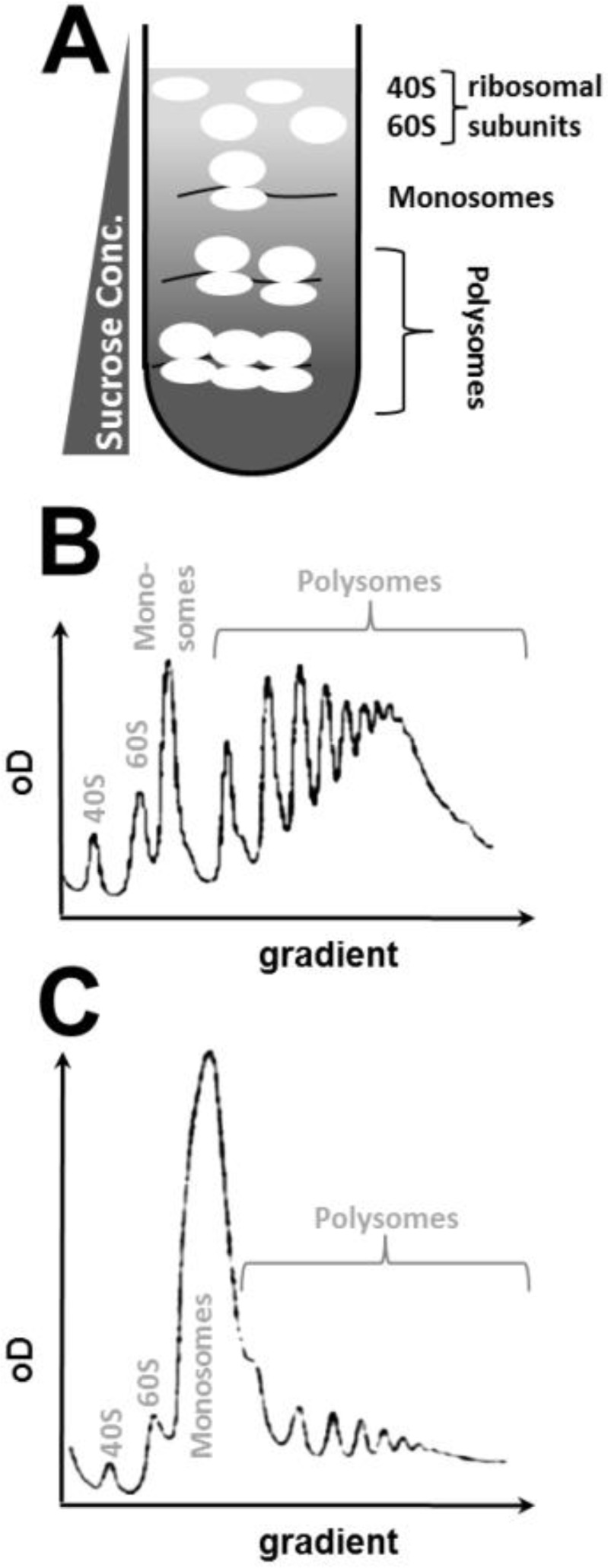
**Polysomal gradients as a tool for studying translational activity. A**, principle of density gradient fractionation. Cell extracts are layered onto density gradients formed with sucrose or glycerol and centrifuged at high speed. Denser structures such as polysomes penetrate more deeply into the gradient than monosomes or free ribosomal subunits. **B and C**, o.D. scans of actual polysomal gradients obtained with actively growing (B) or translationally impaired (C) yeast cells.

The problem of predicting polysome features from translational rate constants, or of extracting rate constants from observed polyribosome profiles (Figure 2), immediately generated substantial interest among theoretical biologists and mathematicians. Since the 1960s, this has led to a continuous flow of publications attempting to provide mathematical and computational solutions for determining ribosome position, ribosome density, protein synthesis rates, and similar parameters as a function of translation initiation, elongation, and termination rates.

In the past, modelling studies only ever constituted a minor fraction of the enormous numbers of publications generated by the very active protein synthesis field. The success of Systems Biology as a new sub-discipline in the life sciences has increased the trickle of modelling studies to a solid river, and it is likely that this will increase to a torrent in the not too distant future. A (near-) comprehensive overview of relevant studies will soon be no longer possible. This review was written with two aims in mind: 1), to serve as a reference repository for studies that have been conducted up to the current date, and 2), to present an overview over approaches, concepts and results as an entry point for those wishing to become familiar with this field.

In modern terminology, all of the older and many of the newer studies come under the “bottom-up” type of systems biology [6], which aims to understand the complex behaviour of a larger system from the known, often simple behaviour of its components. Recently, more and more studies have also employed the opposite “top-down” approach to studying translation, i.e. the reconstruction of the rules governing translation from large-scale observations of the system [7–9]. This review is concerned solely with the “bottom-up” type of approaches.

## Analytical approaches to modelling translation

### ODE-based approaches

The re-writing of chemical reaction systems as systems of ordinary differential equations (ODEs) is straightforward, and probably one of the most commonly used modelling approaches [10]. Since protein synthesis consists of a series of standard biochemical reactions, such an approach is feasible in principle also for the process of mRNA translation. However, due to the cyclical nature of the elongation process and the fact that multiple elongation processes can occur on a single template, arriving at appropriate ODE structures is not as straightforward as for most other enzyme systems.

Typically, in ODE-based models of translation each possible codon:ribosome complex is described as an independent chemical species, and the progression of a ribosome from one codon to the next (or from the *i*th to the *i + 1*th codon) is treated as a first-order chemical reaction *R*_*i*_ *→R*_*i + 1*_ . Thus, an mRNA of length *l* requires *l-1* ODEs to describe all possible elongation steps, plus additional ODEs for the initiation and termination reactions. If an elongation step is to be more realistically presented as a series of reactions, rather than a single reaction, separate ODEs must be introduced for each reaction to be modelled.

It is possible to accurately describe polysome structures with this approach by introducing different species for every possible combination of ribosome occupancies, thus for example denoting an mRNA containing two ribosomes on the fourth and tenth codons as *R*_*4*_ *R*_*10*_. However, the number of ODEs required then approaches (*l-1*)^*d*^, where *d* is the maximum density of ribosomes on the modelled mRNA. Moreover, this type of model introduces a number of artefacts: for example, it inherently assumes that ribosome occupancy is not limited to one per codon, but can be multiple or fractions of one. In consequence, ribosomes in these types of models do not impede each other's progress when they collide.

Despite the numbers of ODEs involved, Gerst and Levine [11] used a Laplace-transform approach to solve an ODE system for an RNA of 6 codons, which could accommodate up to two ribosomes at a time (the actual size of ribosomes was unknown at the time). Considering the size of the equation system even for this simple case, it is clear why analytical solutions based on ODEs are rarely used. However, the ideas from this paper were later modified to derive descriptions for the incorporation of radioactive label into newly synthesized protein, a widely used experimental technique [12]. ODE-based models also often form the basis for computer-based numerical analyses (see below).

### Statistical approaches

As an alternative to the deterministic ODE-based models, approaches based on the statistical properties of ribosome movement were explored initially by Zimmerman and Simha [13, 14], and then refined by MacDonald and Gibbs [15, 16]. These groups considered mRNAs as lattices on which ribosomes move with specific hopping probabilities, the latter being functions of the intrinsic kinetics of elongation; the ratio of initiation, elongation and termination rates (which determine the ribosome density on the message); and the probability that progress of a ribosome is unimpeded by preceding ribosomes (which is itself a function of the ribosome density on the message).

The statistical approach proved popular in a number of modelling studies which modified the basic solutions provided by MacDonald and Gibbs for the investigation of specific questions, such as competition between messenger RNAs with different initiation rate constants [17–20], genome-wide translation systems in *E. coli* [21, 22] and ER-associated translation of secretory proteins [23].

### TASEP-based approaches

The statistical approach as developed by MacDonald and Gibbs [15, 16] continued to be developed theoretically and eventually became known as the “Totally Asymmetric Exclusion Process” or *TASEP*. Despite its origin in attempts to describe ribosome movement along mRNAs, *TASEP*-based approaches were initially not widely used to address protein synthesis problems. Instead, they enjoyed significant success in analyses of vehicular road traffic flows [24]. From there, they found their way back into biology, and the last ten years have seen an explosion of *TASEP*-based studies of translation [25–39].

Characteristics of early versions of the *TASEP* include assumptions of limitless ribosome-supply, a single, uniform elongation rate-constant along the mRNA, and a coarse-grained description of the elongation process, which is simply regarded as a “hopping-probability”. Recent modifications to the basic *TASEP* allowed analyses of codon-specific elongation rates [26–28, 30, 33], limiting supplies of ribosomes [31, 37, 38] or tRNAs [35], and traffic on circularised mRNAs [25], thus making the approach more physiologically relevant. Several recent studies also described approaches that go beyond the description of ribosome movement as a simple hopping probability, and instead consider the detailed sub-steps of the translation elongation cycle [29, 32, 33, 36, 39].

### Other approaches

In addition to the approaches described above, specialised approaches were developed to address specific questions on the process of translation. For example, Krakauer and Jansen modelled the effect of opposing demands on codon usage by the need to optimise host cell translation while “dis-optimising” translation of parasitic (eg viral) genes [40]. Their results led them to propose that the evolution of host and parasite codon usage shows characteristics of “Red Queen Dynamics”, i.e. a race in which the competitors constantly stay abreast with each other but can never gain an advantage.

Other studies have used specialised mathematical models to analyse the effect of mRNA decay on polysome shape [41–44], to analyse the effect of highly expressed heterologous mRNAs on rare tRNA availability [45], to quantify differences in selective pressure between synonymous codons [46], to determine in how far the avoidance of nonsense errors contributes to natural selection between synonymous codons [47], and to quantify the effect of frame-shift errors on translation [48]. All of these models are largely based on statistical analyses of the behaviour of ribosomes on mRNAs.

## Computational approaches to modelling translation

While analytical solutions can yield meaningful descriptions of the behaviour of ribosomes, the theory on which they are based is usually difficult for non-specialists. Richard Gordon, one of the earliest proponents of computer simulations in the study of translation, noted that in his analyses *“... the exact solution for polysomes carrying no more than two ribosomes is exceedingly cumbersome, and that larger polysomes are essentially intractable*” [49]. In Gordon's opinion, computer simulation was “*a more fruitful and highly general approach*”.

Computer simulations are generally used in two ways, both requiring knowledge of rate constants or rates for the individual reactions that form the model. The first approach is to establish systems of ordinary or stochastic differential equations that describe every reaction of the modelled process. These ODE or SDE systems are then used to compute numerical approximations of the development of the system over time, for a given set of parameters and starting conditions. Although this approach is sometimes used to model the actual movement of ribosomes on mRNAs [50–53], it suffers from the same difficulties as outlined above for analytical models of translation regarding the size of equation systems that can result from describing each possible codon:ribosome complex as an individual species. However, numerical approximations have been the main approach for modelling the sub-processes of translation initiation [54–57] and termination [58].

The second approach is to use information on the rates of individual reactions of translation to “animate” the movement of ribosomes on mRNAs one reaction at a time. In this Monte Carlo approach, randomly generated numbers are used to a) select one reaction from all the reactions possible in the system, and b) decide whether or not this reaction will proceed, by comparing the random number to a probability value derived from the rate of the selected reaction. This approach has been used to simulate ribosome movement on mRNAs [49, 59–66], as well as the stochastic tRNA sampling process underlying translation elongation [67]. The average results from many individual Monte Carlo simulations converge exactly on the average behaviour of the described system, but this approach is computationally expensive for large systems.

A specific variant of Monte-Carlo simulations are so-called agent-based models [10, 68]. In these types of model, the modelled particles such as ribosomes or mRNAs are represented by individual variables, rather than pools of particles as in non-agent-based approaches. This has several advantages, including the possibility of tracking the life-history of an individual particle, and the possibility of making the variables “state-rich”: for example, a ribosome might be modified by phosphorylation events that modulate its individual rates or rate-constants. It is noteworthy that the very first models of mRNA translation used this type of data structure, and thus constituted rudimentary agent-based models long before this term was in use [49, 59].

## Model scopes

Initial models of translation typically comprised a single mRNA to which ribosomes attached with a fixed rate *υ*_*I*_. Ribosomes then moved along the mRNA in one-codon steps with a single rate *υ*_*E*_, before being released from the last codon with a rate *υ*_*T*_ [17, 49, 59, 60] (Figure 1). Inherent assumptions arising from the formulation of these models included an unlimited supply of ribosomes and tRNAs, as well as a codon-independent rate of movement. The complete set of input parameters consisted typically of values for the three rates, the mRNA length, and the number of codons covered by one ribosome.

From these beginnings, models grew with the biological knowledge on one hand, and with computational power on the other. Notable extensions that were introduced include multiple competing mRNA species [18] up to complete, genome-wide transcriptomes [21, 52]; multiple, codon-specific *υ*_*E*_ [62, 64, 66]; and the use of rate constants and species concentrations rather than rates [18, 19].

**Figure 3:**
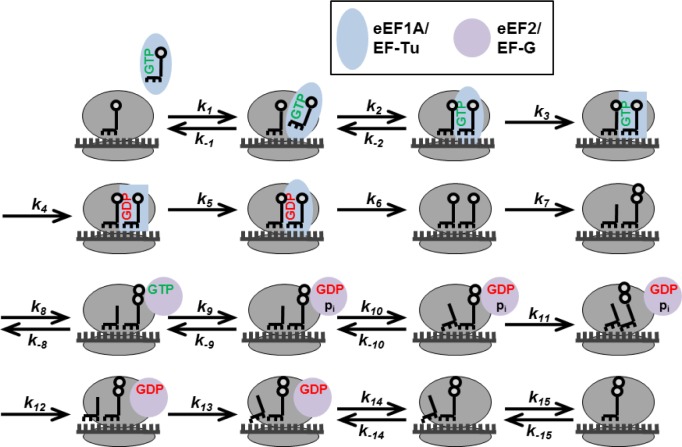
**A fine-grained model of one translation elongation step**. The codon decoding reactions (k_1_-k_7_), and translocation reactions (k_8_-k_15_) are shown with the known, biochemically distinguishable reaction steps (compiled from refs. 67, 51). Shape changes indicate conformational changes in the translation elongation factors. The recycling of the GDP-bound form of EF-Tu/eEF1A is not shown in detail, and tRNA release is shown uncoupled from A-site tRNA binding, although the two reactions may actually be coupled [ref. 91].

The treatment of the three phases of translation as “black boxes” with a single apparent rate was soon realised as being physiologically unrealistic. This led to the development of more fine-grained models, in which one or more of the translational phases were assumed to proceed in multiple sub-steps (Figure 3). Thus, Godefroy-Colburn and Thach described initiation via five sub-reactions [20], Heyd and Drew dissected the elongation step into seven sub-reactions [51], and de Silva *et al*. presented several fine-grained descriptions of the termination step [58]. All of these models analysed the full ribosome cycle. Even more fine-grained models were developed for translation initiation (12 reactions, [56]) and elongation (17 reactions, [67]), although these were implemented as stand-alone models that did not consider the respective other two sub-processes.

The selective expansion and collapse of individual sections of translation into more or less fine-grained reaction systems has become a convenient strategy to consider as much detail as is required for any particular analysis, without incurring unnecessary computational cost by modelling everything in detail. This approach has also been extended to include or exclude additional reactions, such as mRNA transcription and decay [41], ribosome-induced peptidyl-tRNA hydrolysis in response to translational errors (“ribosome editing”, [64]), ribosomal slow-down at mRNA secondary structures [61], and aminoacyl-tRNA synthesis [69].

## Results from modelling studies and their impact on experimentalists

Throughout all of the studies cited above, the aims of computational and mathematical biologists have remained remarkably constant and centred around two important themes.

*To aid in the interpretation of experimental data*. The very first modelling studies were conducted with the stated aim of exploring the relationship between polysome profiles and translational rate constants, because it was realised that the relationship between the two was complex, but that the establishment of any defined relationships between the two would be a useful tool for experimentalists.

*To investigate rate-limitations in the process of translation*. Since the first studies on protein synthesis, the search for “the rate-limiting step” of translation was an important problem that attracted interest from theoreticians and experimentalists alike. Although recent results indicate that the control of translation is highly distributed and the idea of a single rate-limiting step is thus likely to be an oversimplification [57, 70], discussions on the role of individual translation factors as rate-limiting or not rate-limiting constitute an important part of the literature on translation [71–77].

Among the most successful findings from modelling studies, at least as judged by the numbers of citations received, was Lodish's prediction that message-specific translational control could be exerted by canonical translation factors [18]. It probably helped the success of this study that the author provided experimental evidence for the correctness of his model-derived predictions in the same paper as the model. Another study by Rapoport et al [23] analysed signal recognition particle (SRP)-mediated pausing during the translation of secreted proteins. The principal findings from this study were that SRPs arrest ribosomes individually (rather than arresting an entire polysome at a time), and that the translational arrest would only have functional consequences under conditions of strongly limiting SRP abundance. These findings were later confirmed experimentally, and remain widely cited.

Initial modelling studies focussed in particular on the question whether initiation or elongation activity limited protein production rates. For individual mRNAs and physiological ratios of initiation to elongation rates, initiation appeared clearly limiting [16]. This view was strongly taken up by the experimental community, and many recent papers still contain general statements referring to translation initiation as *the* rate limiting step of translation. However, later modelling studies clearly showed that relatively small changes to models can transfer control from initiation to elongation and/ or termination [19].

A parameter that is particularly important in this context is the availability of free ribosomes. Formal control analyses showed that as the levels of available (non-translating) ribosomes approach zero, control over cell-wide translational activity is quantitatively transferred from initiation to elongation [19]. This is because under ribosome-limiting conditions, initiation events cannot occur unless translating ribosomes finish synthesizing the last protein and become available for initiation on the next message. Under such conditions, faster or slower average translation elongation rates can significantly control rates of subsequent initiation events.

It is interesting to note that all current computational models envisage that a ribosome which has finished translating an mRNA exchanges with the cytoplasmic ribosomal pool, and selection of the next mRNA to be translated by that ribosome occurs in a strictly stochastic manner. However, recent experimental results show that eukaryotic ribosomes may translate mRNAs in multiple cycles before entering the free ribosome pool [78]. This would affect the control of translation profoundly, and compared with single-cycle models, it could transfer significant levels of control to the elongation stage.

At what level of ribosome depletion control is transferred to elongation depends in complex ways on the codon composition of the genome. The average speed of translation is not only a function of the numbers of slow codons in a message, but also of their distribution [45, 79]. A particular role is played by the first codons following the start codon, which need to be translated in order to physically liberate the start codon for the next initiation event. Slowly decoded codons within the first ten codons of an ORF can significantly modulate translation initiation rates via this mechanism [62].

**Figure 4:**
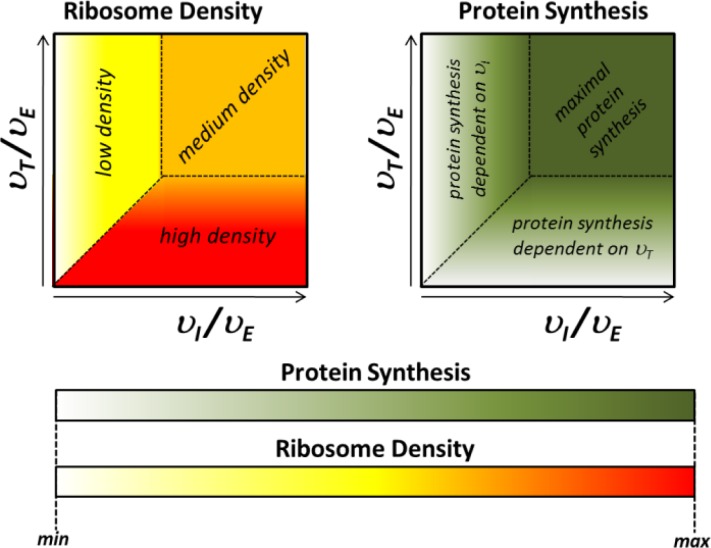
**The relationship between ribosome density and protein synthesis**, as predicted by *TASEP* models. The left panel shows ribosome densities as a function of different ratios of *υ*_*I*_, *υ*_*E*_ and *υ*_*T*_ (initiation, elongation and termination rates) under the standard *TASEP* assumptions of a uniform elongation rate and of unlimited ribosome supply. The right panel shows the corresponding protein synthesis rates.

A particular take on the question of protein synthesis limitations is provided by the *TASEP*-based approaches. Since the earliest studies by MacDonald and Gibbs, it was predicted that different combinations of initiation, elongation and termination rates result in distinct phases of ribosome densities and protein synthesis rates on an mRNA template [16]. Typically, low initiation rates result in low ribosome densities (LD phase), whereas low termination rates result in high ribosome densities (HD phase). High initiation and termination rates compared to elongation rates result in intermediate ribosome densities (MC phase), but this last phase carries the highest protein synthetic capacity. Importantly, the transition between the three phases is predicted to be not gradual but relatively sharp (Figure 4), and the exact location of the phase boundaries for an individual mRNA could thus be an important parameter of translational control. However, it is currently unclear whether the predicted phase transitions occur within physiological parameter limits.

Even if eventually proven physiologically relevant, *TASEP*-derived predictions are likely to paint an overly simplified picture of the different density phases that can occur on an mRNA. One particular simplification is that ribosome cooperativity is seen as exclusively negative (ribosomes may impede each other through collisions). Positive cooperativity could also arise, for example if a first ribosome disrupts secondary structure that then allows a closely following, second ribosome to progress faster through this part of the mRNA. Whether a particular secondary structure generates positive cooperativity depends on the exact parameters associated with it (stability, local ribosome speed preceding and following the structure, mean ribosome density etc). von Heijne *et al*. used stochastic models to investigate ribosome progress through secondary structures, and predicted that under the right conditions, the presence of secondary structure can lead to significantly reduced ribosome passage times with increasing ribosome density [61].

A last example for the use of modelling studies in evaluating features of polyribosomes addresses the long-standing question whether there are systematic changes in the density of ribosomes between the beginning and the end of the message. Several sets of experimental data indicate that ribosomes may be generally more widely spaced, and/ or translate faster, toward the end of an mRNA. The relevant experimental observations include relatively shorter ribosome transit times for larger proteins [80], and higher average ribosome loads [81] or ribosome densities [82] for shorter mRNAs compared to longer ones. However, examination of individual mRNAs has so far failed to identify shifts in ribosome density between the 5’ and 3’ portions of long messages [82, 83]. It is therefore unclear whether the data reflect underlying differences in translation on short and long messages, or differences in translation of 5’- and 3’-ends of the same message.

**Figure 5:**
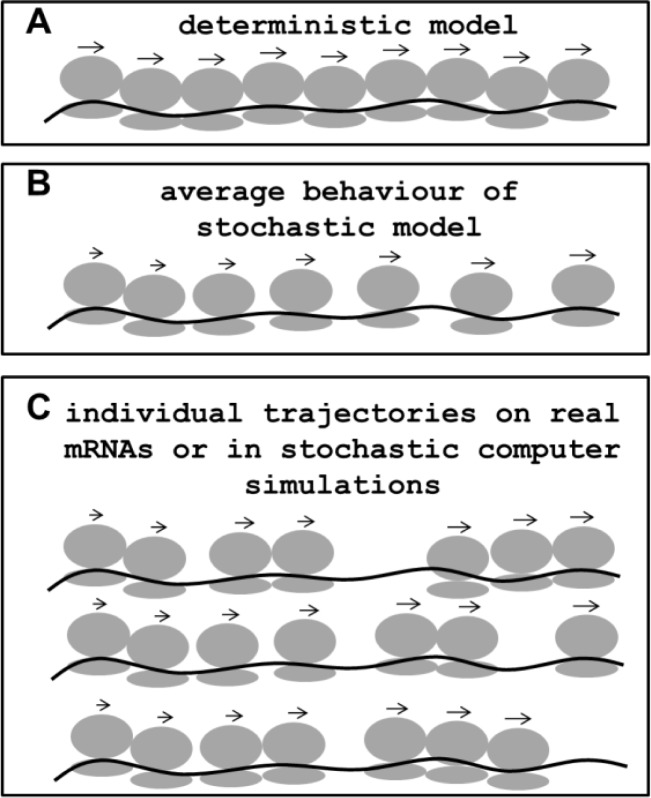
**Illustration of ribosome fluxes on an elongation-limited model mRNA** (*υ*_*E*_ *< < υ*_*I*_, *υ*_*T*_). The Ribosome distribution along an mRNA is drawn as predicted for the simple mRNA illustrated in Figure 1 by a rate constant-based deterministic model (**A**), by analytical models based on stochastic theory (**B**), and as they might be observed on individual, real mRNAs or predicted by stochastic computer simulations (**C**). See text for further explanation.

Several modelling studies have addressed this problem, and propose various potential explanations for the experimental data. Gordon observed that under conditions of dense ribosome packing random fluctuations can lead to an increase in ribosome spacing, even when it is physically impossible to decrease spacing [49]. He reasoned that under such conditions, the net effect of stochastic fluctuations in density would therefore be an increase in ribosome spacing along the message (Figure 5). Ribosome editing, ie the sensing of missense errors by the ribosome and the ensuing premature release of ribosomes, would similarly lead to a random removal of ribosomes from the message that becomes more likely the further the ribosome progresses [64]. Nonsense errors, the erroneous, release-factor induced termination of protein synthesis on sense codons, could have similar “thinning out” effects [47]. Yet further explanations are derived from the observation of translational “speed ramps”, ie stretches of slowly decoded codons immediately following the start codon, which are a conserved feature in many organisms [84].

While it is clear that each of these theories could indeed explain the experimental observations, it is far from clear whether any of these mechanisms do actually contribute to translation and its regulation *in vivo*, and if they do, what their quantitative role is. It is likely that only targeted experiments together with modelling studies will eventually answer these questions.

## Summary and Outlook

In their by now long history, mathematical and computational models have helped to explain experimentally observed features of the translational apparatus. Historically, modelling has been a field that was largely separate from experimentalists. The recent trend for closer integration of models and experiments within the same study [eg 58, 69] is likely to elevate modelling from a specialist discipline to a more widely used tool, and to lead to a true systems biological cycle of experimental data-derived models and model-inspired experiments [85].

Some inspiration for novel types of translational models may come from approaches pioneered for modelling transcription by RNA polymerases, which have significantly advanced understanding of these molecules [86–88]. Some of the concepts developed in that field are highly relevant to the process of translation, but have not yet been applied to the latter. It is notable that recent advances in modelling transcription arose from a close interaction between models and single-molecule experiments, and this experimental technique is now also being applied to ribosomes [89]. The very recent advent of studies that apply engineering tools to analyses of translation and its regulation [90] promises further exciting developments for the future.
